# Advanced MRI in cerebral small vessel disease

**DOI:** 10.1177/17474930221091879

**Published:** 2022-04-20

**Authors:** Hilde van den Brink, Fergus N Doubal, Marco Duering

**Affiliations:** 1 Department of Neurology and Neurosurgery, University Medical Center Utrecht Brain Center, Utrecht University, Utrecht, The Netherlands; 2 Centre for Clinical Brain Sciences, UK Dementia Research Institute, University of Edinburgh, Edinburgh, UK; 3 Medical Image Analysis Center (MIAC AG) and qbig, Department of Biomedical Engineering, University of Basel, Basel, Switzerland

**Keywords:** Cerebral small vessel disease, MRI, quantitative MRI, diffusion tensor imaging, cerebrovascular reactivity, blood–brain barrier, ultrahigh field MRI

## Abstract

Cerebral small vessel disease (cSVD) is a major cause of stroke and dementia. This review summarizes recent developments in advanced neuroimaging of cSVD with a focus on clinical and research applications. In the first section, we highlight how advanced structural imaging techniques, including diffusion magnetic resonance imaging (MRI), enable improved detection of tissue damage, including characterization of tissue appearing normal on conventional MRI. These techniques enable progression to be monitored and may be useful as surrogate endpoint in clinical trials. Quantitative MRI, including iron and myelin imaging, provides insights into tissue composition on the molecular level. In the second section, we cover how advanced MRI techniques can demonstrate functional or dynamic abnormalities of the blood vessels, which could be targeted in mechanistic research and early-stage intervention trials. Such techniques include the use of dynamic contrast enhanced MRI to measure blood–brain barrier permeability, and MRI methods to assess cerebrovascular reactivity. In the third section, we discuss how the increased spatial resolution provided by ultrahigh field MRI at 7 T allows imaging of perforating arteries, and flow velocity and pulsatility within them. The advanced MRI techniques we describe are providing novel pathophysiological insights in cSVD and allow improved quantification of disease burden and progression. They have application in clinical trials, both in assessing novel therapeutic mechanisms, and as a sensitive endpoint to assess efficacy of interventions on parenchymal tissue damage. We also discuss challenges of these advanced techniques and suggest future directions for research.

## Introduction

Cerebral small vessel disease (cSVD) is a major cause of ischemic and hemorrhagic stroke, and vascular cognitive impairment.^1^ It is typically characterized by multiple tissue alterations visible on magnetic resonance imaging (MRI), including but not limited to white matter hyperintensities (WMH), lacunes, cerebral microbleeds, and enlarged perivascular spaces.^2^ These cSVD lesions on MRI can be useful in clinical practice for diagnosis and some, in particular WMH, can be reliably segmented, for example, using deep learning-based algorithms.^3^ However, a lesion-based MRI approach has limitations as the lesion-based concept dichotomizes tissue alterations into normal and abnormal, which does not reflect the gradual-onset of tissue damage found in cSVD.^3^ Quantitative imaging approaches provide a continuous (rather than dichotomized) measure of brain abnormalities, allowing increased granularity in assessment of pathology. In the first section of this review, we highlight quantitative MRI and how these advanced structural imaging techniques enable better assessment of cSVD severity and progression as well as how they provide insights into tissue composition on the molecular level.

Parenchymal lesions are a downstream consequence of vessel pathology and allow only an indirect assessment of cSVD. As such, tissue alterations do not capture early pathological changes in vascular integrity and function, which could be targeted in mechanistic research and early-stage intervention trials.^4^ Recent advances in image acquisition and processing techniques have allowed investigation of vascular function and direct imaging of the small vessels to further elucidate cSVD pathogenesis and to facilitate assessment of prognosis and treatment effects.^5,6^ In the second section of this review, we focus on blood–brain barrier (BBB) permeability and cerebrovascular reactivity. The small vessels themselves are the focus of the third section in which we cover how increased resolution provided by ultrahigh field MRI allows imaging of structure as well as flow velocity in perforating arteries.

### Scope of the review

We aim to summarize the use of advanced MRI in cSVD for clinicians and researchers. We address potential future applications, technical feasibility, status of technical and clinical validation,^7^ and challenges (summarized in Table 1). Details on acquisition and analysis are beyond the scope of this review.

**Table 1.** Technical validation status in cSVD patients, strengths, and weaknesses of advanced MRI techniques.

Technique	Potential applications	Technical validation	Strengths	Weaknesses
Advanced structural imaging				
Diffusion MRI (in particular DTI metrics)	Monitoring disease progression over time, endpoint in clinical trials^8,9^ Prediction of dementia^10^	High scan-rescan repeatability in cSVD patients High inter-site reproducibility when using harmonized acquisition^11^	Widely available and straightforward to implement Short acquisition time when using multiband imaging Fully automated analysis possible (e.g., PSMD)^8^	Especially prone to motion artifacts and CSF contamination^12^ High degree of harmonization needed for comparability across sites^13^
Quantitative MRI (relaxometry, iron, myelin)	Measuring tissue composition^14–16^ and repair^17^	Limited data in cSVD patients	Post-mortem validation^18,19^	Typically needs long acquisition time or research sequences^20^
Cerebrovascular integrity and function				
DCE-MRI	Monitoring disease progression, improve prognosis, personalize medications^21,22^	Limited data concerning repeatability and reproducibility in cSVD patients	Ability to detect small changes in permeability with good spatial resolution	Complicated technique with low signal-to-noise ratio
CVR-MRI	Monitoring disease progression, improve prognosis, personalize medications^21,22^	Limited data concerning repeatability and reproducibility in cSVD patients	Excellent spatial resolution in detection of vascular reactivity Good tolerability	Care needed in image registration. High degree of harmonization needed for comparability across sites
Imaging of small perforating arteries				
Perforating artery morphology and flow velocity	Provide insight in cSVD pathogenesis^23,24^ Potential (treatable) endpoint in clinical trials at the level of the small vessels	Scan-rescan repeatability and inter-scanner reproducibility are topic of ongoing studies	Imaging at the level of small vessel pathology itself Potential to identify small vessel changes before permanent parenchymal damage occurs	Limited availability of 7 T systems Potentially more claustrophobic than 3 T scanner Prone to motion, given the high resolution and relatively long scan time

cSVD: cerebral small vessel disease; MRI: magnetic resonance imaging; DTI: diffusion tensor imaging; DCE-MRI: dynamic contrast enhanced MRI; CVR-MRI: cerebrovascular reactivity MRI; CSF: cerebrospinal fluid; PSMD: peak width of skeletonized mean diffusivity.

### Search strategy and selection criteria

To focus on recent developments, PubMed was searched for articles between January 1, 2018 and November 1, 2021. Titles and abstracts were screened for relevance and full-text of relevant articles reviewed. Further relevant studies were identified from recent reviews.^5,6,25–28^

Search terms used were for cSVD: “cerebral small vessel disease,” “Cerebral Small Vessel Diseases” [MeSH], “small vessel disease,” and microangiopathy. Diffusion MRI: diffusion AND MRI, “diffusion tensor imaging” and “diffusion tensor imaging” [MeSH]. Quantitative MRI: “quantitative MRI,” relaxometry, “magnetization transfer,” “myelin water” and “quantitative susceptibility mapping.” Dynamic contrast enhanced MRI: “dynamic contrast enhanced” AND MRI, “blood brain barrier” AND MRI, “blood-brain barrier” AND MRI, “permeability imaging.” Cerebrovascular reactivity: cerebrovascular reactivity,” “blood oxygen level dependent “, vasodilatation AND MRI, vasoconstriction AND MRI, “carbon dioxide challenge.” Flow velocity imaging: “blood flow velocity” AND MRI, “blood flow pulsatility” AND MRI, 7 T.

## Advanced structural imaging

Quantitative MRI (qMRI) measures physical properties of tissue, supposedly largely independently from the acquisition technique or scanner hardware. In a strict sense, qMRI comprises techniques to estimate relaxation times, called relaxometry, generating quantitative maps for T1, T2, and T2*. In a broader sense, techniques measuring other physical properties are also considered qMRI, such as diffusion MRI as well as iron- and myelin-sensitive acquisitions.

### Diffusion MRI

Diffusion MRI indirectly probes tissue microstructure by quantifying water movement, which appears increased in cSVD.^29^ Most previous work studied metrics from the diffusion tensor imaging (DTI) model. While not specific for a particular pathology, DTI alterations in the elderly seem mostly driven by cSVD and not neurodegenerative pathology, such as Alzheimer’s disease.^30^ In terms of clinical validation, multiple cross-sectional and longitudinal studies have shown strong associations with clinical deficits in cSVD, as highlighted by a recent systematic review.^25^ DTI can detect early tissue alterations even in white matter appearing normal on conventional imaging. The precise assessment of disease burden allows prediction of the clinical course, for example, determining the risk of dementia.^10^ High reliability and high sensitivity to subtle tissue alterations make DTI analysis especially suited to capture change over time. This is best reflected in small sample size estimates for clinical trials using change in DTI metrics as endpoint.^8,9^

Recent studies explore diffusion MRI models more sophisticated than DTI, but they typically require more elaborate acquisition, for example, with sampling of more directions and higher/multiple diffusion-weights, and thus longer scan time. Studies using free water imaging suggested that increased extracellular water is a key factor underlying tissue alterations^29^ and associated with altered hemodynamics.^31^ One study highlighted a benefit of diffusion kurtosis imaging for characterizing the very subtle white matter alterations in early-stage cSVD patients.^11^

**Figure 1.**
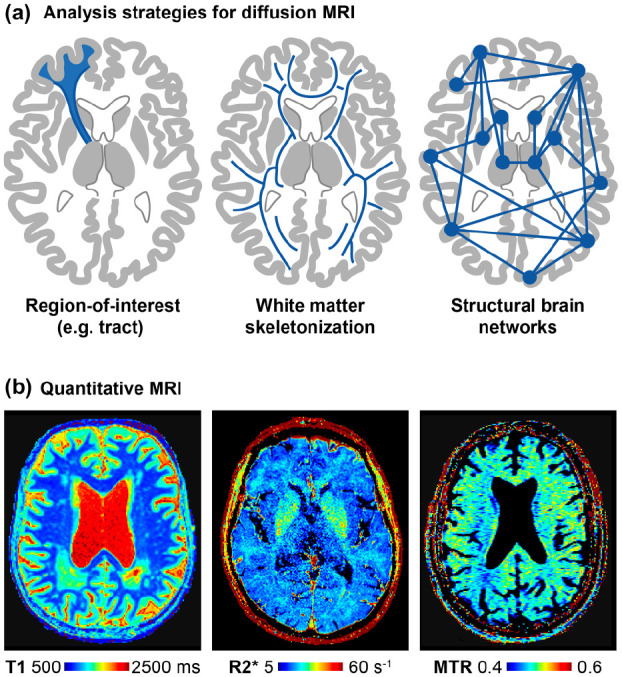
Quantitative MRI: (a) Analysis strategies for diffusion MRI can vary greatly in their complexity. (b) Relaxometry maps for T1 and R2* (1/T2*). Magnetization transfer ratio map as a proxy of myelin content.

There are multiple analysis strategies for diffusion MRI with different levels of complexity (Figure 1(a)). A simple but powerful approach is to study global white matter metrics. This can be fully automated, as in the publicly available peak width of skeletonized mean diffusivity pipeline (PSMD, www.psmd-marker.org).^8^ This fully integrated analysis solution is tailored to cSVD research and easy to implement also in clinical trials.^32^ The most sophisticated analysis approach is to reconstruct structural brain networks using tractography and atlas-based brain parcellations, with analysis of network structure through graph-theoretical metrics. Structural network analysis is much more reliable than (resting-state) functional network analysis in cSVD^33,34^ and can provide pathophysiological insight, such as the importance of central hub connections.^35,36^ However, for quantifying disease burden and progression, the added value of network analysis over simpler analysis approaches seems limited.^37^

### Iron and myelin imaging

Apart from better assessment of disease burden, qMRI enables the study of the molecular composition of tissue. The combination of T1 and R2* relaxometry (Figure 1(b)) has been used to further characterize tissue alterations^14^ and to identify heterogeneity in the composition of white matter hyperintensities depending on location, suggesting different pathophysiology across brain regions.^15^

T2* (or its inverse R2*) mapping is used as a proxy for iron concentration, supported by postmortem validation.^18^ Iron is found in multiple brain cell types and its deposition is considered a marker of degeneration. Quantitative susceptibility mapping also quantifies iron and has been validated for deep gray matter.^19^ Using these techniques, studies have shown increased iron deposition in cSVD,^38^ which was associated with other cSVD markers,^39^ disability,^16^ and regional BBB permeability.^40^ Further studies are needed to explore the added value of iron quantification in cSVD, potentially as a marker of (secondary) neurodegeneration.^41^

Quantifying myelin in cSVD is appealing for mechanistic research and clinical trials, potentially enabling assessment of tissue repair in the form of remyelination. The best-established and still state-of-the-art myelin-sensitive technique is based on magnetization transfer, with first studies in cSVD dating back more than 20 years.^42^ A correlation with myelin has been validated for magnetization transfer ratio by postmortem studies in multiple sclerosis.^43^ Recent studies showed associations between magnetization transfer ratio and gait velocity^44^ as well as cognitive function,^45^ although with limited added value over age. With longitudinal studies suggesting the possibility of cSVD burden regression in some patients,^46^ remyelination is a compelling imaging target that needs to be further established in future studies.

In general, qMRI parameters should be interpreted cautiously, the main issue being lack of specificity. Changes in iron can also affect magnetization transfer ratio and R2*/QSM measurements seem not only determined by iron concentration but also oxygenation state.^47^ Newer developments with potentially higher sensitivity and specificity for myelin are inhomogeneous magnetization transfer^48^ and myelin water imaging.^49^ The latter is based on the short T2 relaxation time of compartmentalized water in myelin sheaths and has been validated postmortem in multiple sclerosis.^17^ Ultimately, the choice of qMRI method needs to be tailored to the application, as sensitivity and specificity vary between techniques.^50^

## Imaging of cerebrovascular integrity and function

This review has focused thus far on parenchymal changes in cSVD, but advanced MRI techniques can also demonstrate functional or dynamic abnormalities of the blood vessels.^51^ There is increasing evidence that endothelial cell dysfunction plays a key role in cSVD pathophysiology, leading to increased permeability of the BBB. This subacute failure of maintenance of homeostasis may lead to damage to and thickening of the basement membrane with accompanying increase in stiffness of blood vessel walls. Measuring this initial leakiness and subsequent vasoreactivity (or stiffness) is a key to understanding this pathophysiological pathway.^51^

### Dynamic contrast-enhanced MRI to assess BBB permeability

Gross brain pathology such as brain tumors or acute stroke lead to a lack of a functioning BBB with extravasation of fluids and contrast agents easily seen on routine clinical brain imaging. The increased BBB permeability in cSVD is of several orders of magnitude smaller and accordingly more difficult to demonstrate, but can be measured with dynamic contrast-enhanced MRI (DCE-MRI). DCE-MRI involves injection of a paramagnetic gadolinium-based contrast agent (preferably a slow bolus injection) with subsequent serial T1-weighted scanning over a period ranging from 20 to 30 min.^6^ Contrast accumulates in the blood and the extravascular, extracellular space. Signal enhancement occurs as contrast agent shortens the longitudinal relaxation times of tissue water.

Following image segmentation, data are analyzed to produce a vascular input function and tissue signal–time curves. Semiquantitative (linear modeling of signal enhancement) or quantitative methods adopting pharmacokinetic methods calculate the blood to brain transfer constant *K*^trans^, a measure of the rate at which contrast agent is delivered to the extravascular space per volume of tissue and agent concentration.^6^ The Patlak model is recommended to assess the pharmacokinetics of signal enhancement.^52^

BBB permeability is increased in cSVD and there is some evidence that the increased permeability is associated with cSVD-related stroke, white matter disease and vascular cognitive impairment.^52–54^ Future studies will address if characterizing BBB permeability allows improved prognostication and/or stratified management, for example developing BBB permeability stabilizing drugs for those with the highest degree of baseline BBB permeability.

### Cerebrovascular reactivity imaging

It is proposed that the leaky BBB leads to vessel stiffness and impaired cerebrovascular reactivity (CVR), contributing to tissue damage seen in cSVD.^51,21^ There are varying methods of provoking vasodilation ranging from noninvasive task-based methods (e.g. visual flicker stimuli), to breath-holding to raise CO_2_ levels and pharmacological methods (administration of acetazolamide). Assessment of CVR with block administration of the potent vasodilator CO_2_ however (at a concentration of 6% for 1–3 min) with blood oxygenation level-dependent (BOLD) MRI produces excellent spatial resolution. Preliminary data in patients post minor stroke and healthy controls suggest good repeatability and reproducibility at 1.5 T and 3 T.^55^ Repeatability though was poorer between days than within day and lower in white than gray matter (due to lower signal-to-noise ratio).^21^ Overall, this technique is highly tolerable, even in older patient populations.^26,21,55^

Lower CVR is associated with worsened WMH in patients following minor stroke.^56^ Baseline CVR may predict subsequent cSVD worsening or may even be used to predict response to certain medications allowing for stratification and personalized medication use.^22,57^ Higher field strength MRI may yield promising results as the change in BOLD signal is larger and more weighted toward the cerebral small vessels,^58^ and because the higher spatial resolution allows for more local CVR assessment.

Measuring BBB permeability with DCE-MRI and CVR with BOLD are complicated techniques—there are several associated pitfalls, largely related to the small effect size and low signal-to-noise ratio but recent advances can alleviate some of the systematic errors. The effect of even small amounts of motion can be mitigated by registration,^59^ care needs to be taken with scan segmentation (with appropriate masking of lesions) and determining the vascular input function. Standardizing scanning across different scanners is possible but needs careful attention to detail including regular quality assurance and scanning of phantoms to identify and minimize signal drift.^22,60^

## Imaging of small perforating arteries

With the development of ultrahigh field imaging at 7 T in humans, exciting new possibilities emerge for cSVD research. With 7 T MRI, we can now image the small vessels themselves *in vivo*. Being able to zoom in to the small vessels will play an important role in developing a better understanding of disease mechanisms.

**Figure 2.**
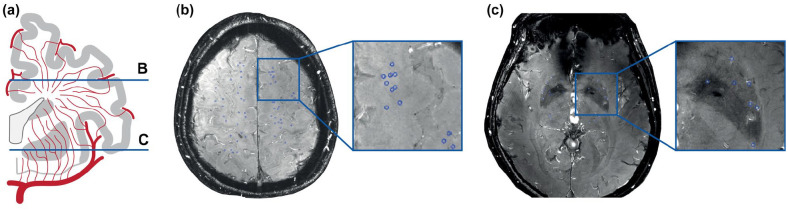
Flow velocity imaging in perforating arteries: (a) Coronal view of perforating artery anatomy. 2D slices in the centrum semiovale (b) and basal ganglia (c) with the perforating arteries marked in blue.

In cSVD, individual lenticulostriate arteries (LSA) were first visualized 12 years ago with 7 T time of flight MRA.^61^ Studies have shown fewer LSA branches in patients with cSVD, previous lacunar stroke and CADASIL compared with controls, which also seemed to relate to cognitive impairment.^61–63^ Over recent years, this study of perforating artery morphology has not translated into morphology-based applications. Instead, in an effort to capture even earlier pathological alterations, focus has shifted toward studying the functioning of these small perforating arteries. Blood flow velocity can now be measured directly in these vessels with 7 T phase contrast MRI. A recent study reported reduced blood flow velocity in LSA in patients with inherited cSVD compared with controls, which was also associated with MRI lesions of cSVD and cognitive function.^23^ From these blood flow velocity measures, a next step is to calculate velocity pulsatility, mostly done with Gosling’s pulsatility index, calculated as (peak systolic velocity–peak diastolic velocity)/mean velocity. Normally, pulsatile blood flow should be dampened as blood travels along the arterial tree, with little remaining pulsatility in arterioles. As already described in the second section of this review, small vessel pathology is hypothesized to cause stiffened vessel walls. Stiff vessels may insufficiently dampen arterial pulse pressure, leading to transmission of higher pulsatility in arterioles where it causes additional damage. Velocity pulsatility measurements in perforating arteries in the basal ganglia and centrum semiovale (Figure 2) in patients with sporadic cSVD indeed showed increased pulsatility compared with controls, despite no difference in flow velocity.^24^ Measuring pulsatility in these small vessels and linking with other dynamic measures such as CVR will help unravel the pathways underpinning cSVD. First efforts on lower field strength show that these assessments can be performed in the basal ganglia with 3 T MRI as well, although with an approximately 5-fold lesser sensitivity and therefore only in the relatively larger perforating arteries.^64^

By imaging at the level of the small vessels, 7 T MRI allows the study of cSVD from a new perspective and potentially captures early pathological changes before more permanent parenchymal damage occurs. Therefore, apart from the need for more validation, the main open question that needs to be addressed in future longitudinal studies is whether *in vivo* small vessel changes in cSVD are merely another consequence of small vessel pathology or causally linked to cSVD parenchymal lesions and cognitive decline. Ultimately, this might enable direct assessment of the effect of new early-stage treatments that target vascular function. With increased installation of 7 T systems and further technical developments, ultrahigh field MRI will undoubtedly play an important role in future cSVD research.

## Conclusion

Advanced MRI contributes to a better characterization of cSVD and has the potential to provide new mechanistic insights. As more complex methods and paradigms are introduced, it is crucial to demonstrate an added benefit over established techniques to justify the increased effort. None of the reviewed advanced techniques is currently in routine clinical use, but diffusion MRI and cerebrovascular reactivity have been used as endpoints in randomized clinical trials. Still, missing technical validation and high instrumental effort are the most apparent challenges for more widespread (clinical) application, which should be the focus of future studies.
